# Infrared Spectroscopy of Bilberry Extract Water-in-Oil Emulsions: Sensing the Water-Oil Interface

**DOI:** 10.3390/bios6020013

**Published:** 2016-04-14

**Authors:** Johannes Kiefer, Kerstin Frank, Florian M. Zehentbauer, Heike P. Schuchmann

**Affiliations:** 1 Technische Thermodynamik, Universität Bremen, Badgasteiner Str. 1, Bremen 28359, Germany; f.zehentbauer@uni-bremen.de; 2 Erlangen Graduate School in Advanced Optical Technologies, University of Erlangen-Nuremberg, Erlangen 91052, Germany; 3 School of Engineering, University of Aberdeen, Aberdeen AB24 3UE, UK; 4 Section I: Food Process Engineering, Institute of Process Engineering in Life Sciences, Karlsruhe Institute of Technology, Karlsruhe 76131, Germany; heike.schuchmann@kit.edu; 5 Present affiliation: BASF SE, Ludwigshafen 67056, Germany; kerstin.frank@basf.com

**Keywords:** MCT oil, hydrogen bonding, interfacial layer, vibrational spectroscopy, functional food, anthocyanin

## Abstract

Water-in-oil (w/o) emulsions are of great interest in many areas of the life sciences, including food technology, bioprocess engineering, and pharmaceuticals. Such emulsions are complex multi-component systems and the molecular mechanisms which lead to a stable emulsion are yet to be fully understood. In this work, attenuated total reflection (ATR) infrared (IR) spectroscopy is applied to a series of w/o emulsions of an aqueous anthocyanin-rich bilberry extract dispersed in a medium chain triglyceride (MCT) oil phase. The content of the emulsifier polyglycerin-polyricinoleat (PGPR) has been varied systematically in order to investigate whether or not its concentration has an impact on the molecular stabilization mechanisms. The molecular stabilization is accessed by a careful analysis of the IR spectrum, where changes in the vibrational frequencies and signal strengths indicate alterations of the molecular environment at the water/oil interface. The results suggest that adding emulsifier in excess of 1% by weight does not lead to an enhanced stabilization of the emulsion.

## 1. Introduction

Emulsions are multiphase systems comprising of two or more liquids that are immiscible with each other. Typically, the *dispersed* phase in the form of small droplets is evenly distributed in the *continuous* phase. To stabilize the emulsion and to avoid macroscopic phase separation, emulsifiers in terms of surface-active substances (surfactants) are admixed. These surfactants are normally soluble, predominantly in one phase, but they exhibit functional groups that can interact with the other phase at the interfacial boundary. On the one hand, the emulsifier represents an additive, which ensures emulsion stability. On the other hand, its concentration should be limited to a minimum in order to save costs and to avoid undesirable effects. For example, in the area of food technology, the emulsifier may alter the taste when its concentration reaches a certain threshold. Hence, in order to optimize emulsion systems there is a need to understand the mechanisms at the interface between the dispersed and the continuous phase. This is the key to develop strategies for selecting the best emulsifier for a given system and minimize the required concentration.

An important step towards understanding the molecular mechanisms is the development of suitable analytical techniques offering high sensitivity for the interactions at the interface. Vibrational spectroscopic methods have proven their capability for studying intra- and intermolecular interactions in pure substances and mixtures [1,2,3]. They have also been successfully applied to emulsions. Jorgensen *et al.* [4] used transmission-mode FTIR spectroscopy to study structural changes of proteins in w/o emulsions. The same method was employed by Zhou *et al.* [5] and Valero *et al.* [6] to study water structures in AOT/alkane/water micro-emulsions of different composition. An attempt to distinguish between interfacial and bulk water IR signals in AOT reverse micelles was made by Sechler *et al.* [7]. They carried out a detailed analysis of the spectra recorded in emulsions with a systematically varied ratio between interfacial and bulk water by varying the droplet diameters. Kemsley *et al.* [8] used attenuated total reflection Fourier transform infrared spectroscopy (ATR-FTIR) to quantitatively analyze the fat content of cream samples. Nickolov *et al.* [9] applied the same technique to investigate water structures in a nanoparticle synthesis which was realized in emulsified droplets acting as micro-reactors. They focused on structural changes inside the droplets during the precipitation process.

A few years ago, our group extended the ATR-FTIR approach to the analysis of water in the interfacial layer of water-in-oil (w/o) emulsions [10]. Making use of the difference in absorption between the oil and the water phase in a selected range of the spectrum allowed the nature of the interfacial molecular interactions to be analyzed. The OH vibrational stretching modes were utilized as sensors for the molecular environment. It was found that the emulsifier weakens the hydrogen bonding network in the interfacial water layer leading to a stabilized water droplet and a decreased tendency of water droplets to coalesce.

In the present work, we apply the above ATR-FTIR method to study the effect of varying the emulsifier content on the molecular stabilization mechanism. The samples under investigation are of interest in the area of food technology and food chemistry: an emulsion where an aqueous anthocyanin solution in terms of a bilberry extract is dispersed in a medium chain triglyceride (MCT) oil stabilized with polyglycerine-polyricinoleat (PGPR) as emulsifier. Anthocyanin compounds are among the most important hydrophilic plant pigments [11,12]. They are considered as food additives owing to their beneficial anti-oxidant, anti-carcinogenic as well as immune modulating effects [12,13,14,15]. Unfortunately, anthocyanins exhibit relatively low chemical stability. Environmental stresses such as pH-values above 4, thermal stress, and the presence of oxygen or certain enzymes accelerate their degradation [16,17]. This poses a challenge in the processing of anthocyanin-containing foods. One possibility to overcome this problem and to protect the bioactive ingredients against environmental stresses is the formulation of multiple emulsions, e.g., a water-in-oil-in-water (w/o/w) system. In such a multiphase system, the sensitive molecules are incorporated into aqueous droplets with a stable chemical composition and pH value for an optimal anthocyanin stability [18,19]. The water droplets are then dispersed in a continuous oil phase. In a further step, this w/o emulsion is dispersed in another aqueous phase. It is needless to say that the formulation of a stable w/o/w emulsion requires both experience and a good understanding of the interfacial phenomena. Despite the effort in the production, such multiple emulsions can offer benefits beyond the mere protection of bioactive ingredients. For example, during the digestive process, the multiple emulsion structure of several cladding layers composed of a triglyceride phase and several emulsifiers and further stabilizing ingredients can enable a triggered release of the encapsulated molecules at different locations in the gastrointestinal tract. Previous work has shown that oil droplets can sustain stomach conditions [20,21]. This makes them a perfect shell for anthocyanin protection in the gastric passage. It was also shown that the protective effect of the capsule structure and a possible targeted release of the encapsulated matter at the effective location strongly depends on the emulsion microstructure [22].

The present study focuses on the initial w/o emulsion system, as the interface between the aqueous anthocyanin solution and the oil is crucial for the behavior from a functional food point of view. The aim of this work is to investigate the effect of the emulsifier concentration on the molecular stabilization mechanism. For this purpose, w/o emulsions are studied and the emulsifier concentration in the oil phase is varied between 1% and 10% by weight, which represents the practically relevant range. The samples are analyzed using ATR-FTIR spectroscopy, for which we have shown previously that the main signals originate from only a thin water layer (below 0.5 µm) at the w/o interface [10]. In addition, we present a vibrational spectroscopic analysis of the individual components of the emulsion system.

## 2. Materials and Methods

### 2.1. Sample Preparation

Anthocyanin-enriched w/o emulsions were prepared as follows. *Aqueous phase:* For the preparation of the aqueous anthocyanin solutions, 0.05% (*w/w*) anthocyanin-rich bilberry extract (Kaden Biochemical GmbH, Hamburg, Germany) was dissolved in distilled water under constant stirring for 30 min via a magnetic stirrer at room temperature. Insoluble solid extract particles were separated by filtration (Sartorius Stedim biotech Germany, Filter Discs, Grade 388). *Oil phase:* The emulsifier-containing oil-phase was prepared by dissolving 1, 5, or 10 wt % polyglycerin-polyricinoleat (PGPR 4150, Palsgaard^®^, Juelsminde, Denmark) in MCT-oil (Medium Chain Triglyceride, Schuman und Sohn GmbH, Germany). The samples were stirred for 1 h via a propeller mixer at 40 °C. *Emulsion:* The w/o emulsions were then prepared via a high pressure homogenizer (MF 110 EH-30, Microfluidics^®^, Westwood, MA, USA) operated at 1000 bar pressure difference, where the water phase (30 wt %) was emulsified in the surrounding oil phase (70 wt %). The diameter of the water droplets in the emulsion was about 2 µm (Sauter mean diameter).

### 2.2. ATR Spectroscopy

The measurements were carried out on a Bruker Vertex v70 FTIR spectrometer equipped with a diamond ATR accessory. The number of reflections at the surface is 1, and the reflection angle is 45°. Spectra were recorded taking 16 scans with a nominal resolution of 1 cm^−1^. All spectra were corrected for water vapor and carbon dioxide interference. In order to check the reproducibility of the measurement, three spectra of each sample were recorded and subtracted from each other pairwise. This subtraction resulted in statistical noise across the entire spectrum. From this, we concluded that statistical errors in the data can be considered to be negligible.

**Figure 1:**
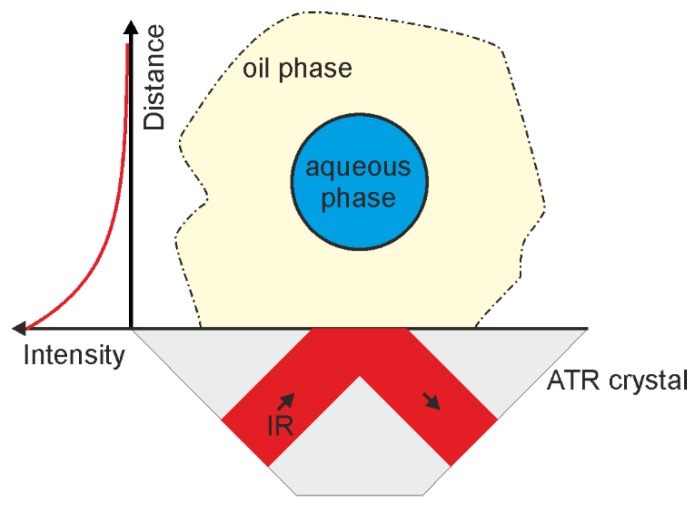
Schematic of the measurement principle. The infrared (IR) radiation is propagating in the attenuated total reflection (ATR) crystal. It undergoes total internal reflection at the surface, where the crystal is in contact with the emulsion sample. The intensity distribution of the evanescent field is illustrated as a function of distance from the surface.

To carry out a measurement, a droplet of the emulsion was placed on the diamond crystal and covered with a cap. The basics of studying w/o emulsions with ATR-IR spectroscopy were described in detail in [10] and Figure 1 shows the situation near the crystal surface. The surface of the ATR crystal is predominantly wetted by the oil phase as no significant contribution from bulk water was observed in the emulsion spectra. On the left hand side of the drawing in Figure 1, the schematic intensity distribution of the evanescent field is plotted as a function of distance from the surface. It represents a single-exponential decay in the absence of absorbing molecules. When a wavelength is considered at which significant absorption in the oil phase takes place, the penetration depth is reduced starting at the surface. On the other hand, when the wavelength of interest is absorbed in the water phase only, the initial intensity decay at the surface remains the same as in the figure, but at the w/o interface the decay constant changes abruptly and the decay is enhanced inside the droplet. As a simplified picture, this can be considered as the product of two exponential functions, one taking into account the medium without absorption and one taking into account the absorption effects via the Beer-Lambert law.

In our previous work [10] it was shown that the resulting thickness of the interfacial water layer that is probed is only a few hundred nanometers. This means that the signals are dominated by the interfacial water rather than from bulk water. Moreover, the probed thickness in terms of its 1/e value is independent of the distance of a droplet from the surface. Therefore, heterogeneities in the emulsion (e.g., in terms of droplet concentration) will not affect the measurement. However, it must be kept in mind that this is only true for the spectral regions where water and not the oil or the emulsifier absorb. Consequently, we focus on the OH stretching region in our analysis.

## 3. Results and Discussion

This section will present the results and their interpretation. We start with the vibrational analysis of the individual components of the emulsions and then proceed to the emulsion samples.

### 3.1. Vibrational Analysis of the Individual Components

#### 3.1.1. Bilberry Extract Solution

**Figure 2:**
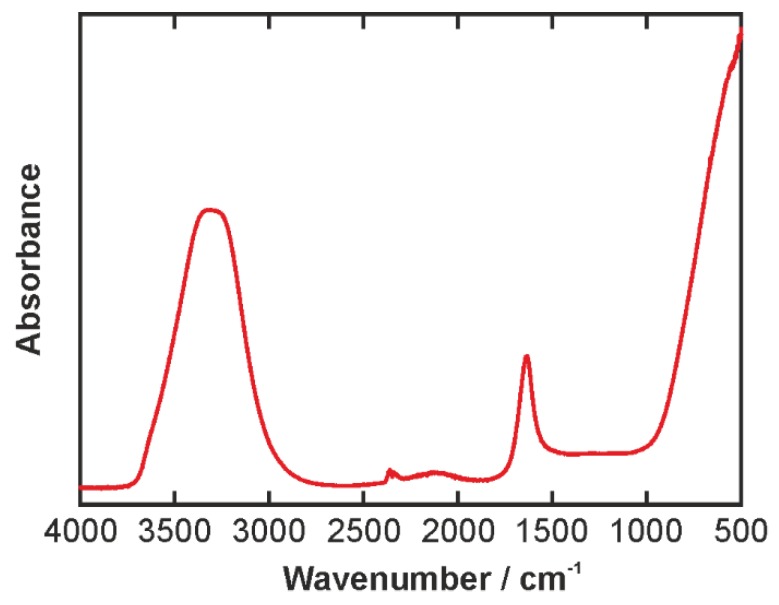
IR spectrum of aqueous bilberry extract solution. The small peak around 2300 cm^−1^ is an experimental artifact of the diamond crystal involved in the ATR measurement.

The IR spectrum of the aqueous solution of the bilberry extract is shown in Figure 2. Due to the very small content of bilberry extract, the spectrum is virtually identical to the spectrum of pure water. It is dominated by broad bands, where the most prominent features are the OH bending mode at 1636 cm^−1^ and the broad OH stretching band ranging from 2800 to 3700 cm^−1^. Our focus will be on the stretching band, the shape of which is an indicator of the hydrogen-bonding environment. In other words, we utilize water as a molecular sensor for studying the effects of the more complicated oil and emulsifier molecules.

For a detailed analysis, the OH bands recorded in the bilberry extract and the emulsions are de-convolved. The de-convolved spectrum will be presented in Section 3.2, but the results for the aqueous bilberry extract are discussed in the following. The OH stretching band was fitted by a sum of six individual Gaussian profiles using a least squares fit algorithm. The fitting parameters were the line position, the line width, and the line intensity. The number of profiles was fixed to allow an interpretation in line with Schmidt and Miki [23] and our previous work [10,24,25]. The results of the fitting procedure are summarized in Table 1. The center wavenumbers slightly differ from our earlier emulsion study [10], but it must be noted that the present spectra were recorded on a different instrument. The lines at 3625 and 2950 cm^−1^ together exhibit only about 5% of the total OH band intensity and are therefore not considered in the following discussion. The low wavenumber component at 3117 cm^−1^ can be assigned to tetra-coordinated, fully hydrogen-bonded water. The higher wavenumber components indicate water, which is not fully coordinated and hence exhibits stronger covalent bonds vibrating at higher frequency [3]. The 3230 cm^−1^ mode can be assigned to symmetrically hydrogen-bonded water, the 3375 cm^−1^ mode to asymmetrically hydrogen-bonded water, and the 3522 cm^−1^ mode to weakly hydrogen-bonded water.

#### 3.1.2. Emulsifier

**Figure 3:**
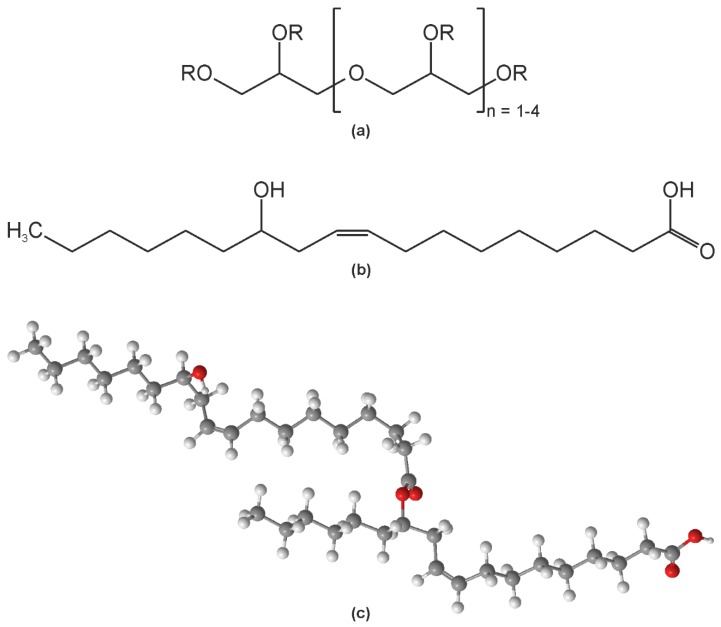
Chemical structure of the emulsifier polyglycerin-polyricinoleat (PGPR). (**a**) General structure where R can be a hydrogen atom or a ricinoleic acid or a polyricinoleic acid substituent; (**b**) Structure of ricinoleic acid; (**c**) 3D model of the structure of diricinoleic acid.

The chemical structure of polyglycerin-polyricinoleat (PGPR) is depicted in Figure 3a. It emphasizes the backbone of polyglycerin and the substituents, R, represent H atoms, or a ricinoleic acid moiety or a polyricinoleic acid moiety. The structure of ricinoleic acid is shown in Figure 3b. It is an unsaturated omega-9 fatty acid and it is the only naturally occurring fatty acid exhibiting a hydroxyl group. This hydroxyl group allows the formation of polyricinoleic acid macromolecules via esterification. Figure 3c shows the 3D sketch of diricinoleic acid as an example. The emulsifier PGPR is strongly lipophilic and virtually not soluble in water at all. However, as it possesses several ether groups and a few OH groups with permanent dipoles it can interact with the water phase and stabilize the droplets by weakening the water-water hydrogen bonds and hence lowering the propensity to coalesce with other droplets.

**Figure 4:**
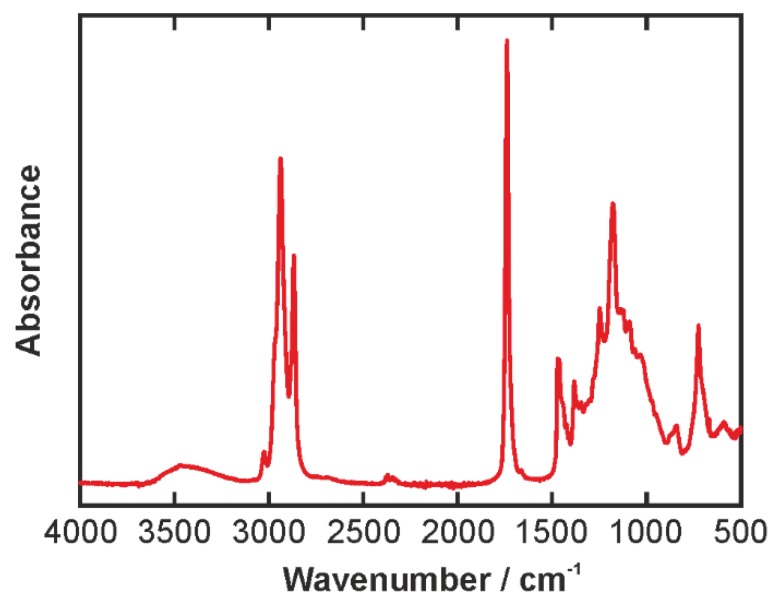
IR spectrum of the PGPR emulsifier. The small peak around 2300 cm^−1^ is an experimental artifact of the diamond crystal involved in the ATR measurement.

Figure 4 displays the IR spectrum of PGPR. The most predominant feature is the C=O stretching mode at 1732 cm^−1^. Further distinct peaks can be found in the CH stretching region of the spectrum around 3000 cm^−1^. Strong peaks are located 2854, 2924, and 2954 (shoulder) cm^−1^, as well as a weaker peak at 3011 cm^−1^. They can be assigned to symmetric and anti-symmetric stretching of CH_2_ and CH_3_ groups. The OH stretching appears as a broad band between 3100 and 3600 cm^−1^. At the low wavenumber wing, the OH stretching band overlaps with the CH stretching peaks, and hence care must be taken in the interpretation of this part of the spectrum. In the fingerprint region, a multitude of mainly CC stretching and CH bending modes is present resulting in a very dense structure of overlapping lines. A detailed analysis of this part of the spectrum would require the aid of computational methods and is beyond the scope of the present study.

#### 3.1.3. MCT Oil and Oil Phase

**Figure 5:**
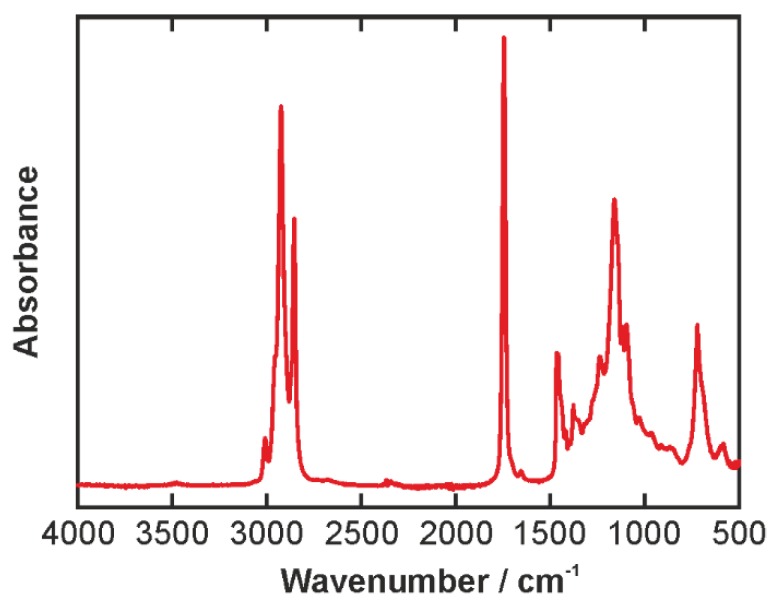
IR spectrum of the medium chain triglyceride (MCT) oil. The small peak around 2300 cm^−1^ is an experimental artifact of the diamond crystal involved in the ATR measurement.

The oil used is a medium chain triglyceride, *i.e.*, a triglyceride typically with aliphatic C6 to C12 fatty acid chains. Figure 5 shows the corresponding IR spectrum. The overall appearance is similar to the emulsifier spectrum discussed in preceding section. The most predominant feature is again the C=O stretching mode, but it is located at 1744 cm^−1^. In other words, it is blue-shifted by 12 cm^−1^ with respect to the band at 1732 cm^−1^ in PGPR. This indicates that the C=O bond in the oils is stronger than in the emulsifier. Further distinct peaks can be found in the CH stretching region at 2854, 2923, and 2956 (shoulder) cm^−1^, as well as a weaker peak at 3007 cm^−1^. Their positions are almost identical compared to PGPR. Again, in the fingerprint region a multitude of mainly CC stretching and CH bending modes is present resulting in a very dense structure of overlapping lines.

**Figure 6:**
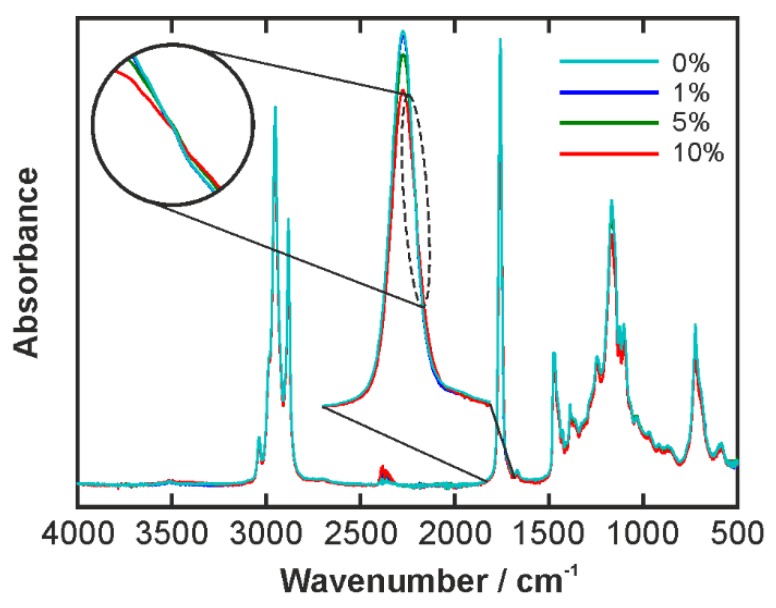
IR spectra of the oil phases containing different amounts of emulsifier. The percentages in the legend are wt % PGPR. The zoomed in region shows the C=O stretching peak. The small peak around 2300 cm^−1^ is an experimental artifact of the diamond crystal involved in the ATR measurement. The zoomed in region in the circle shows the isosbestic point at 1735 cm^−1^.

As aforesaid, the PGPR and oil spectra are very similar due to the structural similarity. Figure 6 shows the IR spectra of the mixtures that were used as continuous phase in the emulsions. They contained 1%, 5%, or 10%wt PGPR. The spectra are very similar as a consequence. The pure oil spectrum is shown as a reference. Distinct changes with the varied emulsifier concentration can be seen at a closer look only. For this purpose, a zoom in of the oil C=O peak around 1744 cm^−1^ is shown in Figure 6 as well. It becomes clear that the peak absorbance is reduced systematically with increasing emulsifier content. Moreover, aside from the change in the peak strength, the zoomed spectrum indicates a small shift as the oil peak at 1744 cm^−1^ decreases and, at the same time, the PGPR peak at 1732 cm^−1^ increases. This results in the appearance of an isosbestic point at 1735 cm^−1^, see inset in Figure 6.

### 3.2. Spectroscopy of Emulsions

**Figure 7:**
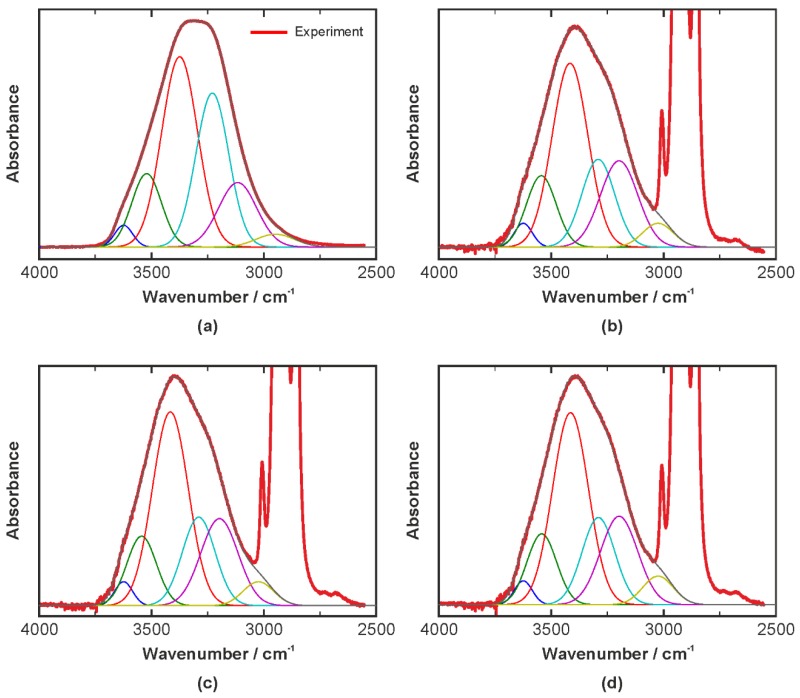
IR spectra of the emulsions in the OH stretching region and the fitted Gaussian profiles. (**a**) Aqueous bilberry extract solution; (**b**) Emulsion with 1% PGPR; (**c**) Emulsion with 5% PGPR; (**d**) Emulsion with 10% PGPR.

The de-convolved OH bands of the aqueous bilberry extract solution and the three emulsions with varied emulsifier content are displayed in Figure 7. As described in Section 3.1.1, six individual Gaussian profiles have been used to fit the band. In each panel of Figure 7, the thick red line shows the experimental data, the thin brown line represents the sum of the Gaussian profiles, which are displayed in various colors individually. In order to get an overview the Table 2, Table 3 and Table 4 compare the center frequencies, the peak widths, and the percentage contribution of the individual Gaussian profiles.

In the emulsions, the OH band and its contributions represented by the Gaussian profiles change significantly, which is in agreement with our previous study [10]. Aside from the peak at 3625 cm^−1^, which exhibits very low intensity in all cases, the peaks are blue-shifted in the emulsions with respect to the aqueous solution, see Table 2. This means that the corresponding covalent OH bonds are strengthened, which in turn indicates that the entire hydrogen-bonding network in the probed water layer at the interface is weakened by the interaction with emulsifier molecules. The peak widths in Table 3 reveal no or negligible differences between the aqueous solution and the emulsions. The same is true in terms of the percentage contribution to the overall band for most of the peaks, see Table 4. Only Peaks 4 and 5, corresponding to the profiles centered at 3230 and 3117 cm^−1^ respectively in the aqueous solution show a significant alteration. The peak at 3230 cm^−1^, which was assigned to symmetrically hydrogen-bonded water, is reduced in the emulsion by approximately one third with respect the aqueous system. On the other hand, the 3117 cm^−1^ peak, which was assigned to tetra-coordinated water, gains about 50% at the same time. This indicates that the fraction of fully hydrogen-bonded water molecules increases in the emulsion. However, it must be kept in mind that the frequency of the peak is blue-shifted with respect to the aqueous solution case. Hence, the data indicate that the network of these fully hydrogen-bonded molecules is significantly weaker than in bulk water.

An interesting finding arises from the data in the Table 2, Table 3 and Table 4, as the changes of the spectroscopic parameters with the variation of the emulsifier concentration can be considered negligible. This indicates that the molecular stabilization at the w/o interface is rather independent of the emulsifier concentration. In other words, adding emulsifier in excess of 1% seems to not lead to an improved emulsion stability. From an engineering and an economic point of few, this finding is rather important as it indicates that a low amount of PGPR is sufficient to stabilize the w/o emulsion.

## 4. Conclusions

In the present work, the vibrational spectra of an aqueous bilberry extract solution, a polyglycerine-polyricinoleat emulsifier and a medium chain triglyceride oil were studied by IR spectroscopy. Based on the vibrational analysis, the IR spectra of a series of water-in-oil emulsions comprising of the above components were studied. The emulsifier concentration was varied systematically. The OH stretching vibration band was utilized as a sensor for the molecular interactions at the water-oil interface leading to a stabilization of the emulsion. A detailed analysis of the OH band revealed that changing the emulsifier content has negligible effects on the stabilization of the emulsion. This is at least true for the concentration range studied, *i.e.*, 1%–10%wt. However, in all emulsion spectra, the individual contributions to the OH stretching band are significantly blue-shifted. In other words, the hydrogen-bonding network in the water phase is weakened as a result of the presence of the emulsifier.

Overall, we can conclude that ATR-IR spectroscopy is a useful tool for studying w/o emulsions. In particular, the OH stretching region of water in the spectrum represents a highly sensitive means of sensing changes in the molecular environment.

## Figures and Tables

**Table 1:** Fitting parameters of the aqueous bilberry extract solution spectrum. Center frequency, peak width, and the percentage intensity with respect to the overall band by peak area are given for the six individual Gaussian profiles.

Profile	Center Wavenumber in cm^−1^	Width in cm^−1^	Intensity in %
peak 1	3625	94	2.24
peak 2	3522	155	12.48
peak 3	3375	188	39.27
peak 4	3230	172	29.07
peak 5	3117	200	14.14
peak 6	2950	200	2.80

**Table 2:** Center frequencies in cm^−1^ of the six individual Gaussian profiles obtained from fitting the infrared OH bands of the aqueous solution and the three emulsions.

Profile	Aqueous Solution	Emulsion with 1% PGPR	Emulsion with 5% PGPR	Emulsion with 10% PGPR
peak 1	3625	3625	3625	3624
peak 2	3522	3544	3544	3542
peak 3	3375	3416	3416	3414
peak 4	3230	3291	3290	3290
peak 5	3117	3198	3198	3198
peak 6	2950	3025	3025	3025

**Table 3:** Peak widths in cm^−1^ of the six individual Gaussian profiles obtained from fitting the infrared OH bands of the aqueous solution and the three emulsions.

Profile	Aqueous Solution	Emulsion with 1% PGPR	Emulsion with 5% PGPR	Emulsion with 10% PGPR
peak 1	94	94	94	94
peak 2	155	155	155	155
peak 3	188	188	188	188
peak 4	172	172	172	172
peak 5	200	195	195	195
peak 6	200	160	160	160

**Table 4:** Percentage intensity with respect to the overall band by peak area of the six individual Gaussian profiles obtained from fitting the infrared OH bands of the aqueous solution and the three emulsions.

Profile	Aqueous Solution	Emulsion with 1% PGPR	Emulsion with 5% PGPR	Emulsion with 10% PGPR
peak 1	2.24	2.68	2.64	2.61
peak 2	12.48	13.23	12.61	12.76
peak 3	39.27	41.34	42.61	41.90
peak 4	29.07	18.06	17.80	17.41
peak 5	14.14	20.14	19.85	20.00
peak 6	2.80	4.55	4.49	5.32

## Abbreviations

The following abbreviations are used in this manuscript:

PGPR: PolyGlycerine-PolyRicinoleat
MCT: Medium Chain Triglyceride
IR: InfraRed
FTIR: Fourier-Transform InfraRed
ATR: Attenuated Total Reflection

## References

[1] Kiefer J., Noack K., Bartelmess J., Walter C., Dörnenburg H., Leipertz A. (2010). Vibrational structure of the polyunsaturated fatty acids eicosapentaenoic acid and arachidonic acid studied by infrared spectroscopy. J. Mol. Struct..

[2] Noack K., Leipertz A., Kiefer J. (2012). Molecular interactions and macroscopic effects in binary mixtures of an imidazolium ionic liquid with water, methanol, and ethanol. J. Mol. Struct..

[3] Joseph J., Jemmis E.D. (2007). Red-, blue-, or no-shift in hydrogen bonds: A unified explanation. J. Am. Chem. Soc..

[4] Jorgensen L., Van de Weert M., Vermehren C., Bjerregaard S., Frokjaer S. (2004). Probing structural changes of proteins incorprated into water-in-oil emulsions. J. Pharm. Sci..

[5] Zhou G.W., Li G.Z., Chen W.J. (2002). Fourier-transform infrared investigation on water states and the conformations of Aerosol-OT in reverse microemulsions. Langmuir.

[6] Valero M., Sanchez F., Gomez-Herrera C., Lopez-Cornejo P. (2008). Study of water solubilized in AOT/n-decane/water microemulsions. Chem. Phys..

[7] Sechler T.D., DelSole E.M., Deak J.C. (2010). Measuring properties of interfacial and bulk water regions in a reverse micelle with IR spectroscopy: A volumetric analysis of the inhomogeneously broadened OH band. J. Colloid Interface Sci..

[8] Kemsley E.K., Appleton G.P., Wilson R.H. (1994). Quantitative analysis of emulsions using attenuated total reflectance (ATR). Spectrochim. Acta.

[9] Nickolov Z.S., Paruchuri V., Shah D.O., Miller J.D. (2004). FTIR-ATR studies of water structure in reverse micelles during the synthesis of oxalate precursor nanoparticles. Colloids Surf. A.

[10] Kiefer J., Frank K., Schuchmann H.P. (2011). Attenuated total reflection infrared (ATR-IR) spectroscopy of a water-in-oil emulsion. Appl. Spectrosc..

[11] Newsome A.G., Culver C.A., van Breemen R.B. (2014). Nature’s Palette: The Search for Natural Blue Colorants. J. Agric. Food Chem..

[12] Tsuda T. (2012). Anthocyanins as Functional Food Factors—Chemistry, Nutrition and Health Promotion. Food Sci. Technol. Res..

[13] Prior R.L., Cao G.H., Martin A., Sofic E., McEwen J., O'Brien C., Lischner N., Ehlenfeldt M., Kalt W., Krewer G. (1998). Antioxidant capacity as influenced by total phenolic and anthocyanin content, maturity, and variety of Vaccinium species. J. Agric. Food Chem..

[14] Cooke D., Schwarz M., Boocock D., Winterhalter P., Steward W.P., Gescher A.J., Marczylo T.H. (2006). Effect of cyanidin-3-glucoside and an anthocyanin mixture from bilberry on ademona development in the Apc(Min) mouse model of intestinal carcinogenesis—Relationship with tissue anthocyanin levels. Int. J. Cancer.

[15] Kong J.M., Chia L.S., Goh N.K., Chia T.F., Brouillard R. (2003). Analysis and biological activities of anthocyanins. Phytochemistry.

[16] Nielsen I.L.F., Haren G.R., Magnussen E.L., Dragsted L.O., Rasmussen S.E. (2003). Quantification of anthocyanins in commercial black currant juices by simple high-performance liquid chromatography. Investigation of their pH stability and antioxidative potency. J. Agric. Food Chem..

[17] Hubbermann E.M. (2005). Functional properties of anthocyanin concentrates and the influence of physicochemical parameters and food additives on the color and stability of isolated anthocyanins in food. PhD Dissertation.

[18] Frank K., Köhler K., Schuchmann H.P. (2011). Formulation of labile hydrophilic ingredients in multiple emulsions: Influence of the formulation's composition on the emulsion’s stability and on the stability of entrapped bioactives. J. Dispers. Sci. Technol..

[19] Baum M., Schantz M., Leick S., Berg S., Betz M., Frank K., Rehage H., Schwarz K., Kulozik U., Schuchmann H.P. (2014). Is the antioxidative effectiveness of a bilberry extract influenced by encapsulation?. J. Sci. Food Agric..

[20] Ax K. (2004). Emulsionen und Liposomen als Trägersysteme für Carotinoide. PhD Dissertation.

[21] Ribeiro H.S., Schuchmann H.P., Engel R., Briviba K., Walz E., Zuidam N.J., Nedovic V.A. (2009). Encapsulation of Caroteniods and Vitamins. Encapsulation Technologies for Food Active Ingredients and Food Processing.

[22] Palzer S. (2009). Food structures for nutrition, health and wellness. Trends Food Sci. Technol..

[23] Schmidt D.A., Miki K. (2007). Structural correlations in liquid water: A new interpretation of IR spectroscopy. J. Phys. Chem. A.

[24] Wallace V.M., Dhumal N.R., Zehentbauer F.M., Kim H.J., Kiefer J. (2015). Revisiting the Aqueous Solutions of Dimethyl Sulfoxide by Spectroscopy in the Mid- and Near-Infrared: Experiments and Car-Parrinello Simulations. J. Phys. Chem. B.

[25] Kiefer J., Grabow J., Kurland H.-D., Müller F.A. (2015). Characterization of Nanoparticles by Solvent Infrared Spectroscopy. Anal. Chem..

